# Insuffisance cardiaque sur cœur normal révélant une malformation artériole-veineuse complexe du membre inferieur chez un enfant: à propos d’une observation et revue de la littérature

**DOI:** 10.11604/pamj.2018.31.131.17078

**Published:** 2018-10-22

**Authors:** Ndèye Fatou Sow, Mohamed Lèye, Idrissa Basse, Yaay Joor Dieng, Mame Aïta Seck, Djénéba Fafa Cissé, Amadou Sow, Mohamed Fattah, Awa Kane, Morgiane Houngbadji, Papa Moctar Faye, Amadou Lamine Fall, Ndèye Ramatoulaye Diagne Guèye, Ousmane Ndiaye

**Affiliations:** 1 Centre Hospitalier National d'Enfants Albert Royer, Avenue Cheikh Anta Diop, Dakar, Sénégal; 2 Clinique de Cardiologie, CHU de Fann, Dakar, Sénégal; 3 Hôpital d'Enfants de Diamniadio BP 204 Bargny, Sénégal

**Keywords:** Malformations artérioveineuses, haut débit, Schöbinger, insuffisance cardiaque, membre inférieur, Arteriovenous malformations, high-flow, Schöbinger, heart failure, lower limb

## Abstract

Les malformations artérioveineuses (MAV) sont des anomalies vasculaires congénitales à haut débit très rares chez l'enfant. Leurs localisations atypiques et leurs manifestations cliniques variables rendent leur diagnostic et leur prise en charge souvent tardifs. Nous rapportons le cas d'un enfant pris en charge au Centre Hospitalier National d'Enfants Albert Royer de Dakar. Un garçon âgé de 9 ans nous a été adressé d'une structure sanitaire en milieu rural pour la prise en charge d'une insuffisance cardiaque. L'examen clinique à l'admission montrait une altération de l'état général, un syndrome d'insuffisance cardiaque globale et une volumineuse masse inguino-crurale droite, chaude, étendue à la paroi abdominale latérale droite (fosse iliaque et flanc droit), à limites mal définies. L'auscultation de cette masse objectivait un thrill et un souffle diffus. L'échographie cardiaque montrait une HTAP sévère avec retentissement important sur les cavités cardiaques, sans atteinte structurelle du cœur. Le diagnostic de MAV a étè confirmé par une échographie doppler de la masse complétée par un angioscanner. Ils mettaient en évidence des fistules artérioveineuses multiples au sein de la masse. Le diagnostic d'une MAV complexe de la racine de la cuisse droite au stade IV de Schöbinger a étè retenu. La prise en charge médicale a consisté au traitement de l'insuffisance cardiaque à base de furosémide, de spironolactone, et de captopril, en vue d'une stabilisation hémodynamique pour une éventuelle cure chirurgicale. Les malformations artérioveineuses des membres, en particulier de l'extrémité proximal du membre inférieur sont encore méconnues chez l'enfant, d'où les erreurs et retards diagnostiques fréquents. Leur évolution est imprévisible d'où la nécessité d'un diagnostic précoce et d'un suivi attentif impliquant une collaboration pluridisciplinaire entre pédiatres, chirurgiens et radiologues.

## Abstract (en)

Arteriovenous malformations (AVM) are congenital high-flow vascular defects. They are very rare in children. Diagnosis and treatment are often delayed due to their atypical place of occurrence and to their variable clinical manifestations. We report the case of a child treated at the National Centre Hospitalier Albert Royer Children, Dakar. A boy aged 9 years was referred from a health facility in rural areas for the treatment of heart failure. Admission clinical examination showed impaired general condition, global heart failure syndrome and voluminous right warm inguinocrural mass extended to the right abdominal wall (iliac fossa and right flank), with poorly defined limits. Auscultation of this mass showed a thrill and diffuse murmur. Cardiac ultrasound showed severe pulmonary arterial hypertension (PAH) with extensive impairement of the heart cavities, without cardiac structural involvement. The diagnosis of AVM was confirmed by doppler ultrasound of the mass complemented by angiography scan. They showed multiple arteriovenous fistulas within the mass. The diagnosis of complex stage IV AVM (according to Schöbinger classification) of the root of the right thigh was retained. Clinical treatment of heart failure was based on furosemide, spironolactone and captopril to obtain hemodynamic stabilization before possible surgical procedure. Arteriovenous malformations of the limbs, in particular of the proximal end of the lower limb in children are still largely unknown, hence frequent diagnostic errors and delays. Their evolution is unpredictable requiring early diagnosis and careful monitoring involving multidisciplinary interaction between pediatrics, surgeons and radiologists.

## Introduction

Les malformations artérioveineuses (MAV) sont des malformations vasculaires rares, dites à haut débit, qui posent des difficultés tant diagnostiques que thérapeutiques chez l'enfant. En effet, elles peuvent être présentes dès la naissance mais demeurer non visibles ou quiescentes, et ne se manifester que tardivement au décours de complications pouvant engager le pronostic fonctionnel ou vital. Nous rapportons le cas d'un enfant dont le diagnostic de MAV complexe de la cuisse droite a été fait au stade ultime de son évolution. A travers ce cas et une revue de la littérature nous discutons les aspects diagnostiques de cette entité.

## Patient et observation

**Figure 1:**
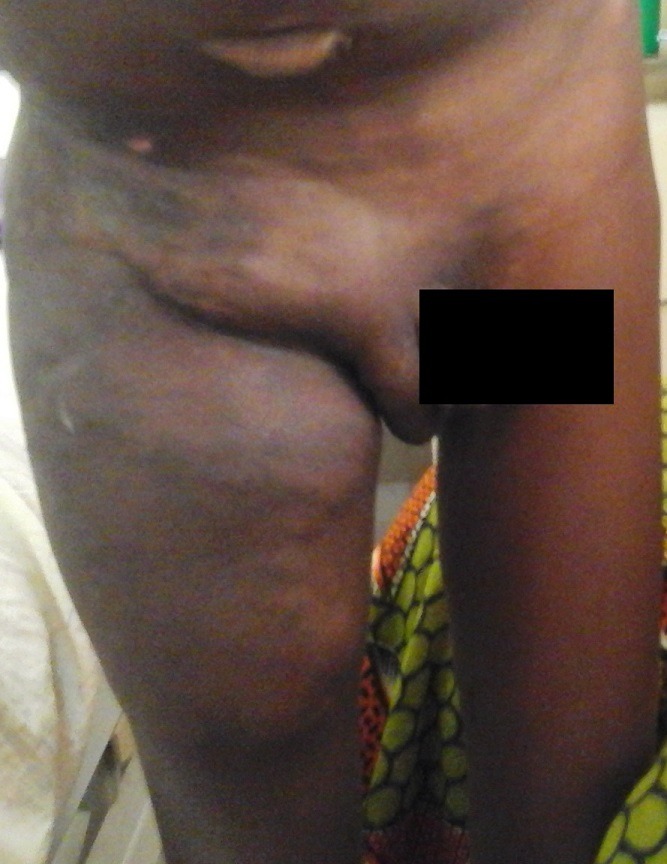
Aspect clinique de la MAV: tuméfaction, tâches bleutées, réseaux veineux tortueux et tendus

**Figure 2:**
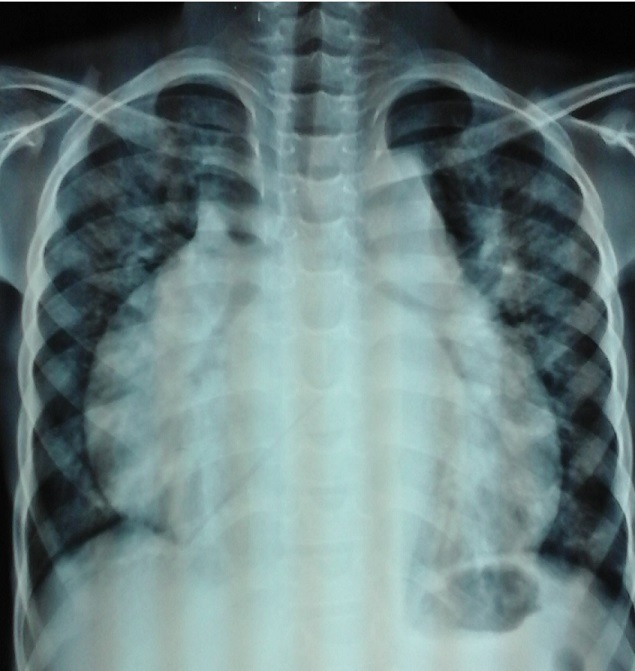
Radiographie du Thorax de Face: cardiomégalie et comblement de l?arc moyen gauche du c?ur

Un garçon âgé de 9 ans nous a étè adressé d'une structure sanitaire en milieu rural pour la prise en charge d'une détresse respiratoire et d'une ascite de grande abondance évoluant depuis 1mois. Il présentait des antécédents chirurgicaux de cure de cryptorchidie bilatérale et de hernie ombilicale à l'âge de 7 ans, et ne présentait aucun antécédent pathologique familial particulier. L'examen clinique à l'admission montrait une altération de l'état général, une insuffisance pondérale (P/A à-3 DS), un thorax en carène et un syndrome d'insuffisance cardiaque globale avec une orthopnée, une tachycardie régulière avec bruits de galop gauche et droit, sans souffle, une turgescence spontanée des veines jugulaires, une hépatomégalie à bord inférieur mousse avec une flèche hépatique de 14 cm associée à un reflux hépato-jugulaire, une ascite libre de grande abondance et des œdèmes bilatéraux des membres inférieurs de type cardiaque. L'examen physique révélait également une volumineuse masse inguino-crurale droite, chaude, étendue à la paroi abdominale latérale droite (fosse iliaque et flanc droit), à limites mal définies (Figure 1). La peau en regard était saine. La palpation objectivait une masse pulsatile et l'auscultation de cette masse révélait un thrill et un souffle diffus. Elle évoluait depuis environs 3 ans, augmentant progressivement de volume. Le bilan biologique était normal. La radiographie du thorax montrait une cardiomégalie importante à pointe sus-diaphragmatique avec un index cardio-thoracique à 0,76, un comblement de l'arc moyen gauche de l'ombre cardiaque et une redistribution vasculaire apicale (Figure 2).

**Figure 3:**
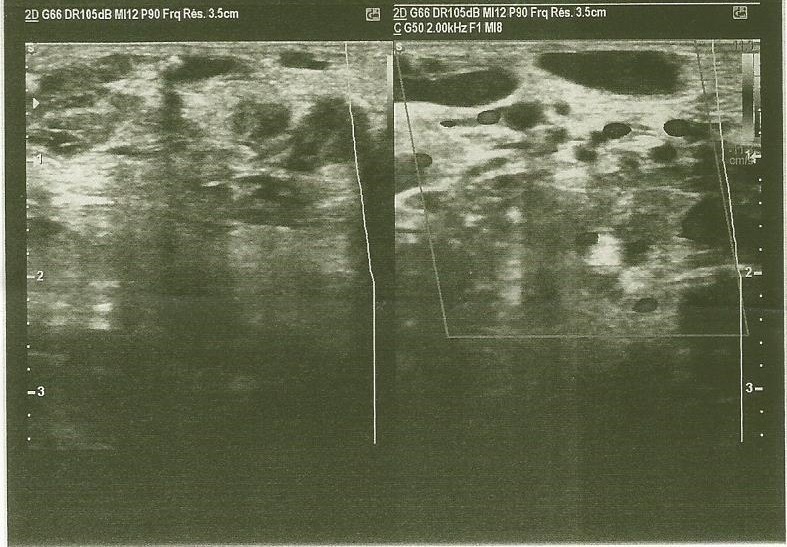
Echographie doppler de la MAV: fistules artério-veineuses (FAV) multiples

**Figure 4:**
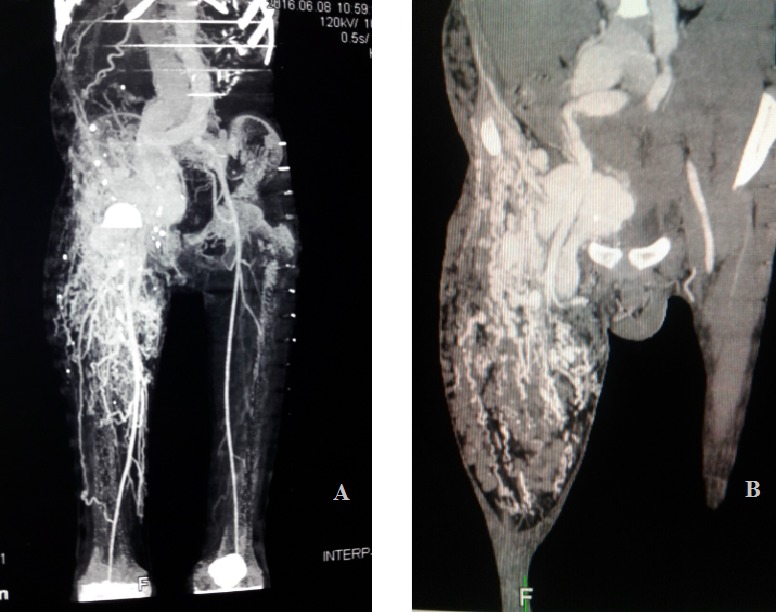
Coupes tomodensitométriques de la MAV

La radiographie standard du bassin de face ne montrait pas de lésions osseuses. L'échographie cardiaque montrait une hypertension artérielle pulmonaire (HTAP) sévère de niveau systémique avec une pression artérielle pulmonaire systolique (PAPS) à 115 mmHg avec retentissement important sur les cavités cardiaques droites, et une dilatation modérée des cavités gauches, sans autres anomalies. L'échographie doppler de la masse mettait en évidence des fistules artério-veineuses (FAV) multiples (Figure 3). L'exploration de la masse à la tomodensitométrie (TDM) objectivait une MAV de 27 cm x 11 cm x 7 cm, occupant la loge antérieure des 2/3 proximaux de la cuisse droite, étendue à la paroi abdomino-pelvienne antérieure droite. Elle présentait plusieurs FAV, sans nidus individualisé et les afférences artérielles provenaient pour l'essentiel des branches fémorales profondes, épigastriques, hypogastriques et fémorales superficielles droites (Figure 4). On notait la présence de quelques phlébolithes sur le versant veineux de la malformation. Le diagnostic d'une MAV complexe de la racine de la cuisse droite au stade IV de Schöbinger était retenu. La prise en charge médicale a consisté dans un premier temps au traitement de l'insuffisance cardiaque à base de furosémide, captopril et spironolactone, en vue d'une stabilisation hémodynamique pour une éventuelle cure chirurgicale. L'évolution sous traitement médical était favorable marquée par une régression du syndrome d'insuffisance cardiaque et le maintien d'une stabilité hémodynamique au bout de 3 semaines. La prise en charge n'avait pu être poursuivie le patient ayant volontairement interrompu le traitement.

## Discussion

Les MAV sont des malformations vasculaires, à haut débit, formées de vaisseaux artériels et veineux dysmorphiques interconnectés directement, sans la transition d'un lit capillaire [1]. Elles sont très rares, représentent globalement 10 à 15% des malformations vasculaires [2] et surviennent à égale fréquence dans les 2 sexes [3]. Il s'agit de lésions congénitales dûes à un défaut de régression des plexus rétiformes primitifs à un stade précoce du développement embryonnaire [4] dont l'origine génétique a été admise par certains auteurs [1]. Ces derniers incriminent un déficit des voies d'apoptose et une dysrégulation des signaux de différentiation vasculaire, mais aussi une mutation du gène *RASA-1*[5]. Toutefois il a étè noté des cas de MAV secondaires à des traumatismes, à des maladies vasculaires dégénératives et des MAV d'origine iatrogène [6,7].

Comme chez notre patient, leur diagnostic peut être souvent tardif car les lésions ne sont pas systématiquement visibles à la naissance. Garzon et al rapportent que les lésions ne sont présentes à la naissance que chez 40 % à 60 % des nourrissons et apparaissent dans l'enfance et l'adolescence dans environ 30 % des cas [1,2]. Enjorlas et al avaient retrouvés cette même variabilité de l'âge de révélation des MAV dans leur étude [8]. Plus particulièrement, les MAV des membres sont décelées en moyenne à l'âge de 2 ans et s'expriment le plus souvent vers l'âge de 11ans [9]. Les localisations aux extrémités proximales des membres comme dans notre cas sont très rares [10]. En effet, les MAV peuvent être de siège ubiquitaire mais les plus grandes incidences sont rapportées à la tête et au cou (50%) [11], au pelvis et aux extrémités distales des membres [12] où elles siègent avec prédilection autour des articulations. Elles sont généralement sporadiques mais quelques cas familiaux sont cependant rapportés [6]. Elles sont le plus souvent isolées mais peuvent apparaitre dans le cadre d'associations syndromiques congénitales comme le syndrome de Klippel Trenaunay où elles siègent essentiellement aux membres inférieurs, le syndrome de Sturge-Weber et la maladie d'Osler Rendu entre autres [6,12]. Le diagnostic des MAV périphériques peut être généralement affirmé à la suite d'un examen clinique mais il est souvent difficile au début car les manifestations sont diverses. Il peut s'agir de lésions cliniquement non visibles, parfois dissimulées dans les muscles, de lésions en apparence innocente sous la forme d'une malformation capillaire[9], ou au contraire de lésions importantes qui peuvent engager le pronostic fonctionnel ou vital. Ces malformations grandissent non par hyperplasie cellulaire mais par des mécanismes hémodynamiques [9]. Schöbinger, en 1995, a proposé une classification clinique pour décrire l'évolution des MAV [4]. Dans le stade I, la MAV quiescente prend l'apparence d'une tache rose, bleutée et chaude. Dans le stade II, la lésion augmente de taille et devient pulsatile. Un thrill est perceptible et les veines de drainage sont tendues et tortueuses comme chez notre patient. Plusieurs auteurs ont retrouvé les mêmes signes [12]. Marshall *et al* [13] avaient, dans leur étude, rajouté parmi les signes les plus caractéristiques une augmentation de la chaleur locale. Le stade III de Schöbinger voit l'apparition de nombreuses complications : signes cutanés dystrophiques, nécrose tissulaire, infections et saignements fréquents, abondants, liés à la fragilité tissulaire. Les douleurs sont persistantes. Une ischémie peut apparaître. On peut également noter comme chez notre patient une déformation inesthétique du membre concerné et une impotence fonctionnelle relative, voire une ischémie distale du membre [14]. Selon Laurian, les complications tissulaires profondes sont représentées par les complications osseuses. Les MAV à gros débit détruisent la corticale osseuse et envahissent la médullaire avec, comme corollaire, la fragilisation osseuse mais tel n'était pas le cas chez notre patient [15].

Le stade IV, ultime stade de l'évolution est celui de la décompensation cardiaque. L'insuffisance cardiaque dans ce cas n'est pas due à une atteinte des structures anatomiques du cœur qui sont saines. Elle est secondaire à une augmentation importante du retour veineux due aux multiples shunts artérioveineux de la MAV et responsable d'une HTAP [14]. C'est à ce stade, que le diagnostic de la MAV a été posé chez notre patient, le tableau d'insuffisance cardiaque étant la principale circonstance de découverte. Cette situation est en réalité extrêmement rare puisqu'elle ne concerne que 1 à 2 % des patients [7]. Elle survient généralement dans les cas de MAV proximales [9], les lésions distales ne présentant pas de shunts suffisamment significatifs pour entraîner une décompensation cardiaque [16]. L'évolution des MAV est donc imprévisible et peut être exacerbée par certains facteurs. Selon Liu et al, les enfants qui ont des MAV stade 1 de Schöbinger présente 43,8 % de risque d'aggravation à l'adolescence [17]. Des auteurs ont trouvé que le débit des MAV pouvait brusquement s'accélérer à la suite de modifications hormonales comme pendant la puberté, de traumatisme, d'infections ou de chirurgies [8,16]. Cela pourra être le cas chez notre patient qui présente des antécédents d'intervention chirurgicale de la région abdomino-pelvienne où les accidents vasculaires iatrogènes sont fréquents du fait de la forte vascularisation [16]. Chez notre patient, le diagnostic a étè confirmé par une échographie doppler de la lésion. L'échographie Doppler pulsé couleur est souvent réalisée en première intention pour confirmer le diagnostic de MAV [2]. Elle repère les points de fistule, évalue les débits et mesure les index de résistance [18]. Elle est non invasive et plus accessible dans notre contexte. Toutefois ce n'est pas le meilleur examen d'imagerie pour l'exploration d'une MAV. L'angio-TDM, que nous avons réalisée chez notre patient offre, par rapport à l'échographie doppler, une meilleure visualisation des structures vasculaires de la MAV et de son extension squelettique ou viscérale. Mais elle reste limitée d'où probablement l'absence de visualisation de nidus dans notre cas. L'angio-IRM est meilleure que la TDM pour l'exploration des MAV. En effet, elle caractérise mieux les artères nourricières et les veines de drainage, mais avec une définition inférieure à une véritable angiographie par cathétérisme [1]. D'ailleurs, Johnson et al ont préconisé de commencer par l'IRM, l'écho doppler ne pouvant explorer les lésions profondes et proches des structures osseuses ou contenant de l'air [19,20]. Mais c'est un examen qui n'est pas toujours accessible dans notre contexte. Nous confirmons donc le diagnostic sur la base de l'écho doppler vasculaire complété de l'angioscanner.

## Conclusion

Les MAV, en particulier celles de l'extrémité proximale du membre inférieur, chez l'enfant sont encore méconnues. La variabilité de l'expression clinique rend le diagnostic peu aisé d'où les errements et retards diagnostiques fréquents. Dans notre contexte le diagnostic repose sur la clinique complétée par l'échographie Doppler. L'évolution imprévisible pouvant mettre en jeu le pronostic vital par une défaillance cardiaque rend leur prise en charge complexe. Celle-ci impose un diagnostic précoce et un suivi attentif, clinique et radiologique, dans le cadre d'une collaboration pluridisciplinaire impliquant pédiatres, chirurgiens et radiologues.

## Conflits d'intérêts

Les auteurs ne déclarent aucun conflit d'intérêts.
